# Gallbladder Carcinoma, the Difficulty of Early Detection: A Case Report

**DOI:** 10.7759/cureus.493

**Published:** 2016-02-12

**Authors:** Andrew E Graff, Stephen L Lewis, Jonathan R Bear, David C Van Echo, Hugh M Dainer

**Affiliations:** 1 Radiation Oncology, Walter Reed National Military Medical Center; 2 Hematology/Oncology, Walter Reed National Military Medical Center; 3 Diagnostic Radiology, Walter Reed National Military Medical Center/Uniformed Services University of Health Sciences

**Keywords:** gallbladder, gallbladder cancer, carcinoma, cancer, cholecystectomy, gallbladder carcinoma

## Abstract

Gallbladder carcinoma (GBC) is an uncommon malignancy with a high mortality rate. Detecting gallbladder carcinoma in its early stages can be difficult, despite improvements in ultrasound and computed tomography (CT) imaging. Most diagnoses of GBC are made at advanced stages, with the majority being found incidentally during surgery for cholelithiasis. The presented case demonstrates the difficulty of diagnosing GBC preoperatively in its early stages.

## Introduction

Gallbladder carcinoma (GBC) is a rare malignancy, but the most common malignancy of the biliary tract. The incidence of GBC in the US is 1.2 per 100,000. The majority of gallbladder carcinomas are diagnosed incidentally, usually when exploring for cholelithiasis [1]. Most diagnoses of GBC are made at advanced stages and have been associated with poor outcomes [2]. Several risk factors have been identified for GBC. The leading risk factors include female gender, cholelithiasis, and advancing age. Gallbladder polyps, porcelain gallbladder, congenital biliary cysts, abnormal pancreaticobiliary duct junction, and carcinogen exposure have all been associated with GBC. Additional reported risk factors include smoking, obesity, diabetes, chronic infections (*Salmonella*, *Helicobacter*), and medications (such as methyldopa, OCPs, isoniazid, and estrogen) [3-5]. Herein, we present a case of locally advanced GBC with node-positive disease found during laparoscopy for acute cholecystitis.

## Case presentation

**Figure 1:**
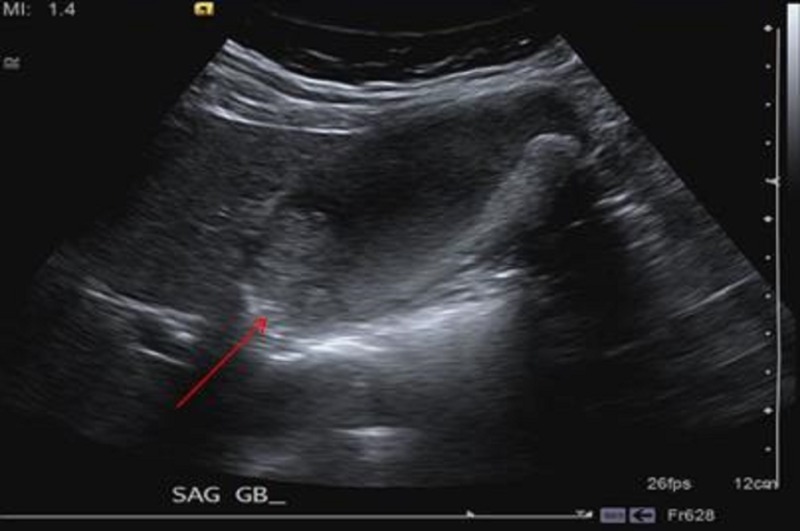
Initial right upper quadrant ultrasound. The image demonstrates intraluminal hypoechoic (red arrow) material. Given the appearance and lack of discrete internal flow, it appears to be most likely due to gallstones or tumefactive sludge, which is a benign mimic of a neoplasm.

**Figure 2:**
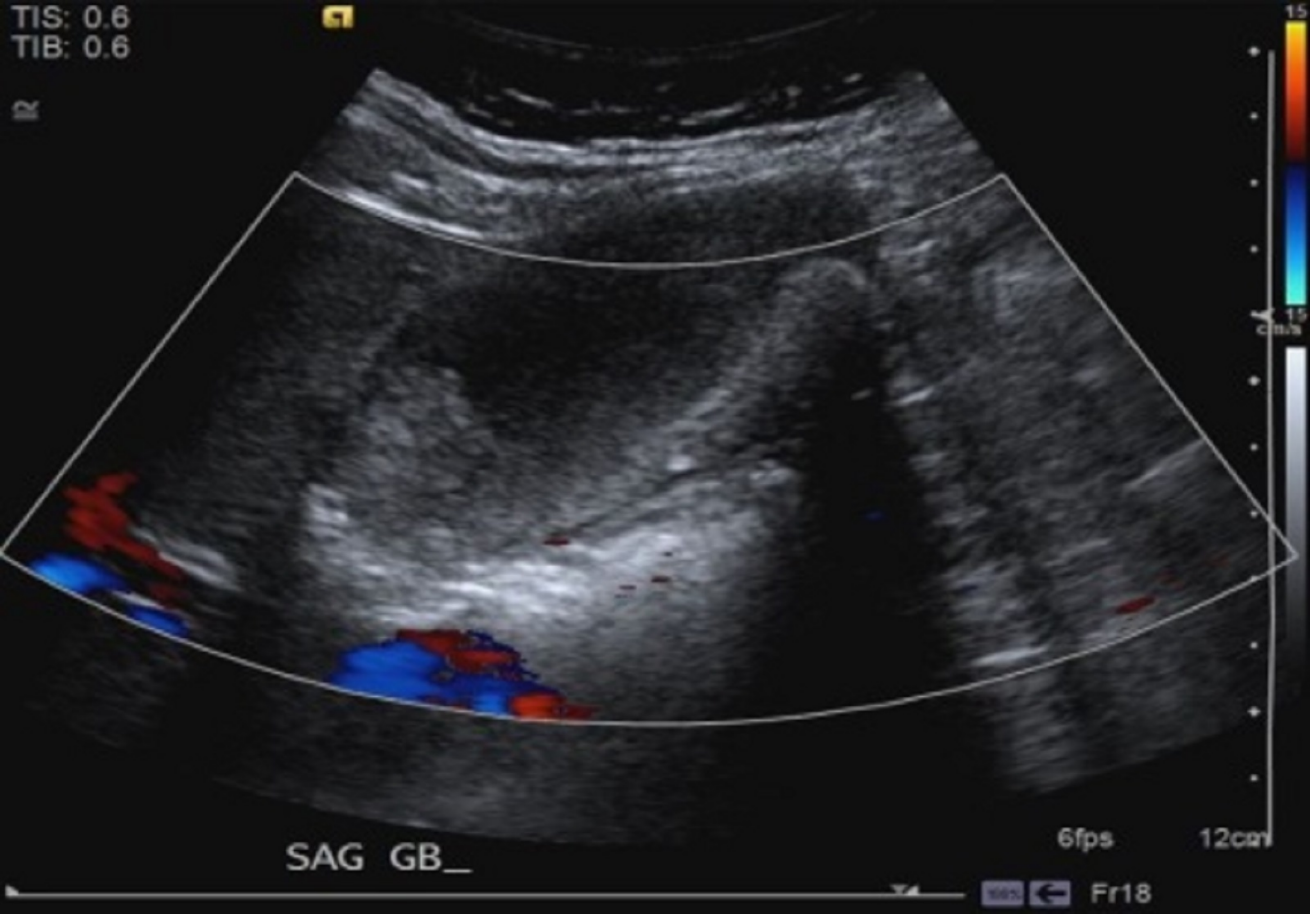
Initial right upper quadrant ultrasound. The image demonstrates no discrete internal flow within the gallbladder with blood flow in the adjacent vasculature.

A 65-year-old obese, African-American female with a history of hyperlipidemia presented to her primary care physician (PCP) in May 2013 for right neck pain radiating to her right shoulder for several weeks. The patient's initial blood work revealed an elevation in her liver enzymes; otherwise, all other lab results were normal. Additionally, a right upper quadrant (RUQ) ultrasound was performed which demonstrated probable gallbladder sludge and stones, with a low likelihood of being polyps or gallbladder neoplasm given the absence of discrete vascularity (Figures 1, 2).

The patient was followed closely by her PCP until her symptoms resolved and elevated liver enzymes normalized over the next few months. No additional imaging was performed at that time, and no surgery was scheduled.

**Figure 3:**
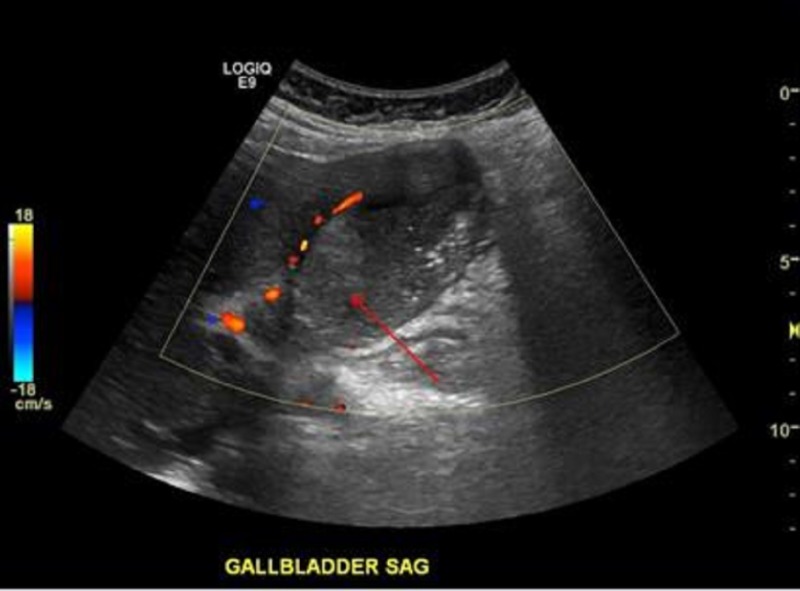
Right upper quadrant ultrasound. The image demonstrates sludge/stone like material within the lumen of the gallbladder (red arrow) with a rim of hyperemia initially interpreted as outside of the gallbladder given the clinical appearance of acute cholecystitis.

**Figure 4:**
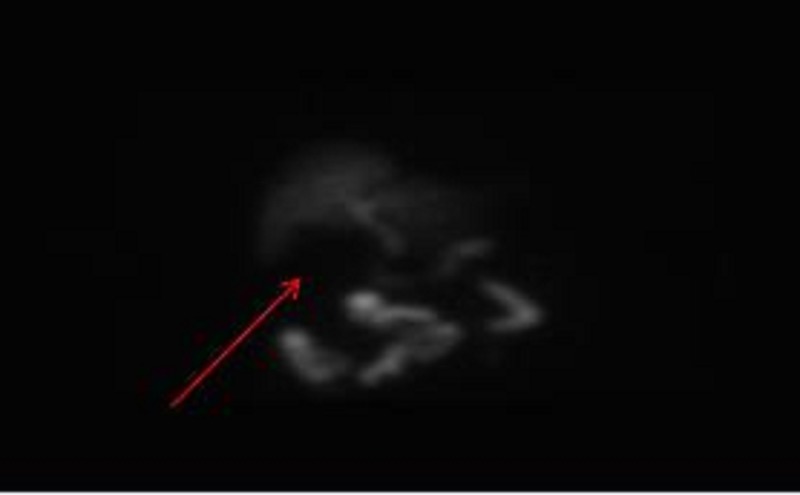
HIDA scan. The image demonstrates excretion through the biliary system into the duodenojejunal junction (DJJ), but with no uptake within the gallbladder/cystic duct (red arrow).

In April 2014, the patient developed RUQ pain that worsened over the next four months, prompting her to seek medical help. In August 2014, she was reevaluated by her PCP and subsequently underwent an acute abdominal series (AAS), RUQ ultrasound, and nuclear medicine hepatobiliary (HIDA) study. The ultrasound demonstrated cholelithiasis and echogenic sludge within the gallbladder with hypoechoic and hyperemic adjacent liver parenchyma. A mildly enlarged common duct was also observed. These findings were concerning for chronic cholecystitis, xanthogranulomatous cholecystitis, or early acute cholecystitis (Figure 3). The HIDA scan results were also consistent with acute cholecystitis (Figure 4).

The patient was taken to surgery for a laparoscopic cholecystectomy for suspected cholecystitis. The laparoscopic approach was converted to an open procedure after the discovery of a mass within the lumen of the gallbladder, a second mass near the hepatic flexure of the colon, and enlarged portal vein lymph nodes. During the operation, the patient underwent a radical cholecystectomy, right hemicolectomy with primary anastomosis, and a portal vein lymph node dissection (LND). Pathology of the gallbladder mass demonstrated infiltrating papillary adenocarcinoma invading the perimuscular connective tissue. The pathology also revealed one portocaval lymph node that was positive for adenocarcinoma. The patient's stage of disease was determined to be IVB (pT2N2). After surgery, the patient underwent radiotherapy to 4500 cGy, targeting the gallbladder fossa and the involved lymph nodes, with concurrent chemotherapy (capecitabine 625 mg/m2 twice daily). Abdominal CT and MRI scans were performed eight months after the completion of chemoradiation. At that time, there was no radiologic evidence of disease recurrence. Currently, the patient is fourteen months post treatment. Overall, she is doing well and continues to receive close follow-up care.

## Discussion

GBC is an infrequent neoplasm that is associated with a high rate of regional lymph node metastasis and mortality [2,6,9]. Adjuvant chemoradiation is commonly recommended for node-positive or incompletely resected disease [7-8]. The extent of the resection correlates with survival. R0 resection is the most important because patients who undergo R2 resections do poorly in spite of chemoradiation. However, there are some long-term survivors in patients treated with adjuvant chemoradiation status post R0/R1 resections [6,9-10]. Therefore, early detection is important because patients found at stage T1N0 would have a greater chance for surgical cure and spare them the potential toxicity of adjuvant therapy.

Early diagnosis can be difficult because symptoms can mimic or be caused by coexisting cholecystitis, which is a common condition [5]. Therefore, screening patients who present with these symptoms is essential given the possibility for a coexisting GBC. Symptoms early in the disease process can also be vague, often leading to a delay in diagnosis. The most common complaint in the symptomatic patient with GBC is RUQ pain, specifically in the right hypochondrium. Other warning signs, which our patient did not exhibit, include weight loss, anorexia, nausea and/or vomiting, jaundice, and pruritus [11-12]. Routine laboratory tests are generally nondiagnostic and do not significantly improve the identification of GBC preoperatively, as exemplified by our case [12]. Serum tumor markers, carcinoembryonic antigen (CEA), and carbohydrate antigen 19-9 (CA 19-9) are frequently elevated in patients with GBC, but are not useful in its diagnosis because of their lack of sensitivity and specificity [13-14].

Imaging with ultrasound and CT has improved preoperative diagnosis of GBC. Despite these advancements, only 50% of gallbladder cancers are recognized before surgery [12]. Ultrasound is often the initial imaging study of choice for patients presenting with symptoms consistent with gallstone disease. The RUQ ultrasound is perhaps the single most important test in helping lead to the diagnosis of GBC preoperatively. Ultrasound images from a group of patients diagnosed with GBC incidentally were reviewed retrospectively and found to have suspicious findings on reevaluation [15].

**Figure 5:**
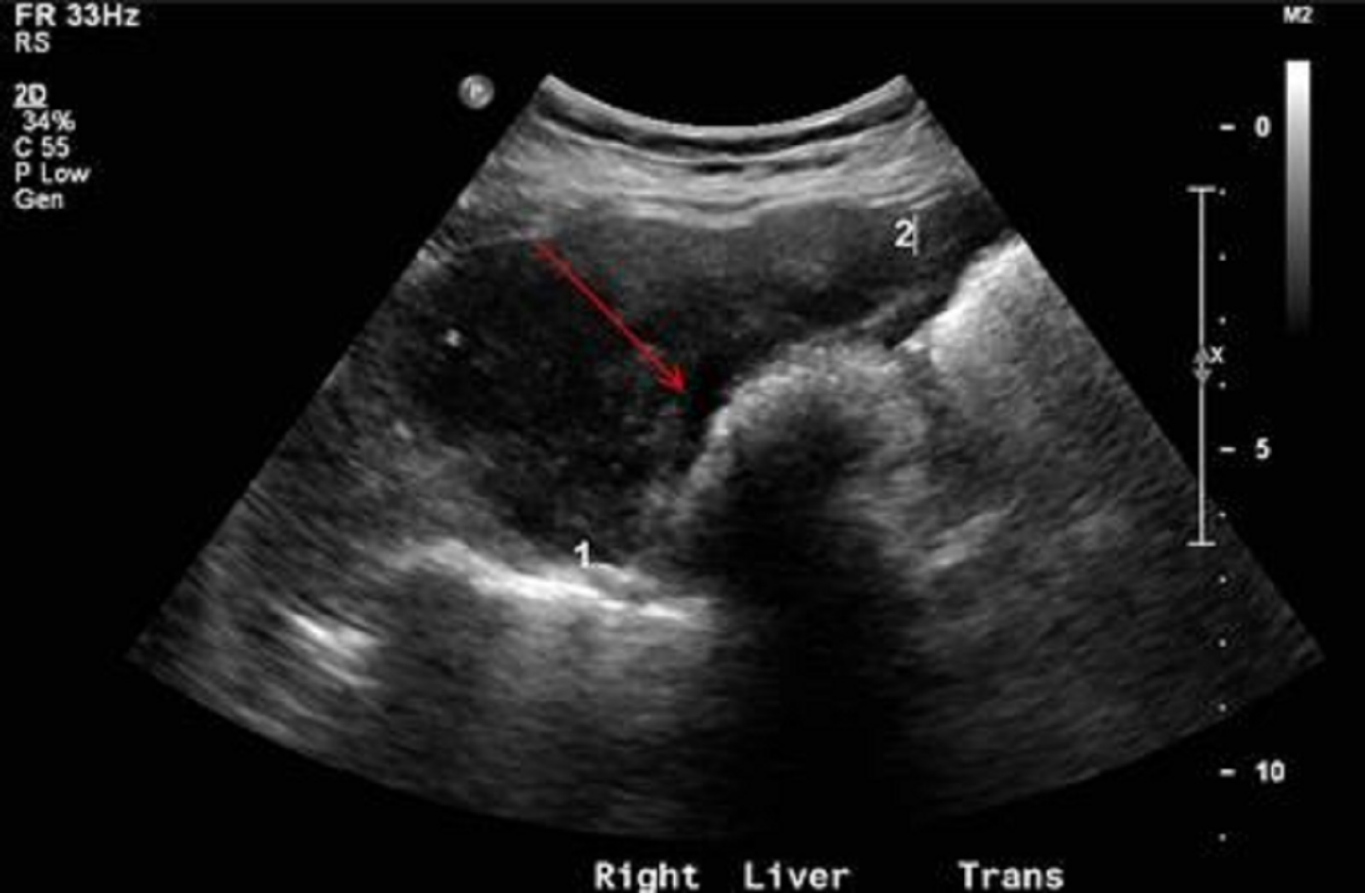
Companion case. Right upper quadrant ultrasound of a patient who presented a few months after the first patient. The image demonstrates a hypoechoic lesion near the expected location of the gallbladder (red arrow). Given the suspicious ultrasound finding and recent prior GBC case, leading differential diagnosis was neoplasm. As a result, further evaluation with a CT scan was recommended.

**Figure 6:**
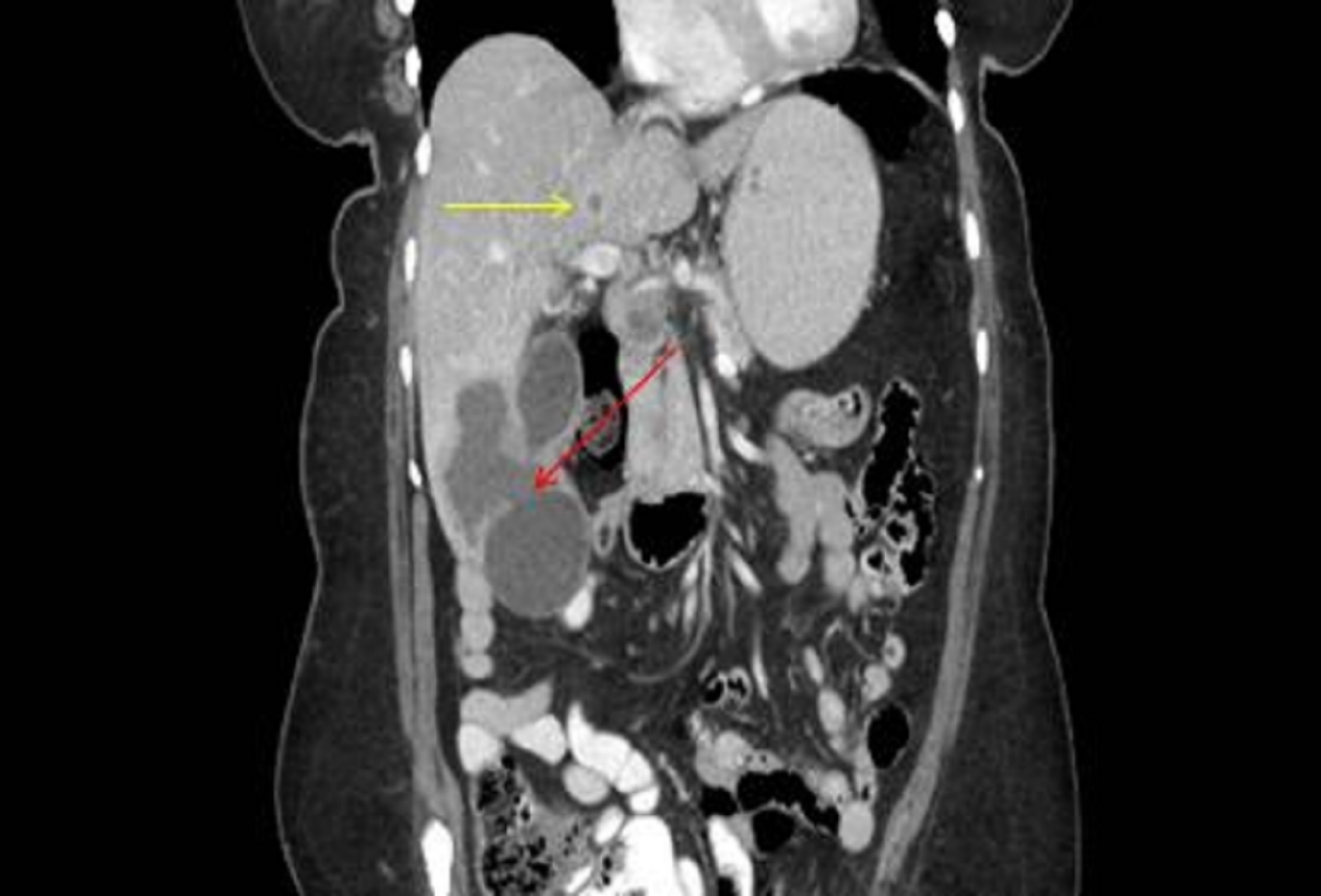
CT scan. The image demonstrates a coronal CT showing a hypodense mass with direct invasion into the right hepatic lobe (red arrow), with additional small lesion in the left hepatic lobe (yellow arrow). Not shown is portohepatous and peripancreatic lymphadenopathy. The extent of disease suggests an element of patient denial of symptoms prior to clinical presentation.

**Figure 7:**
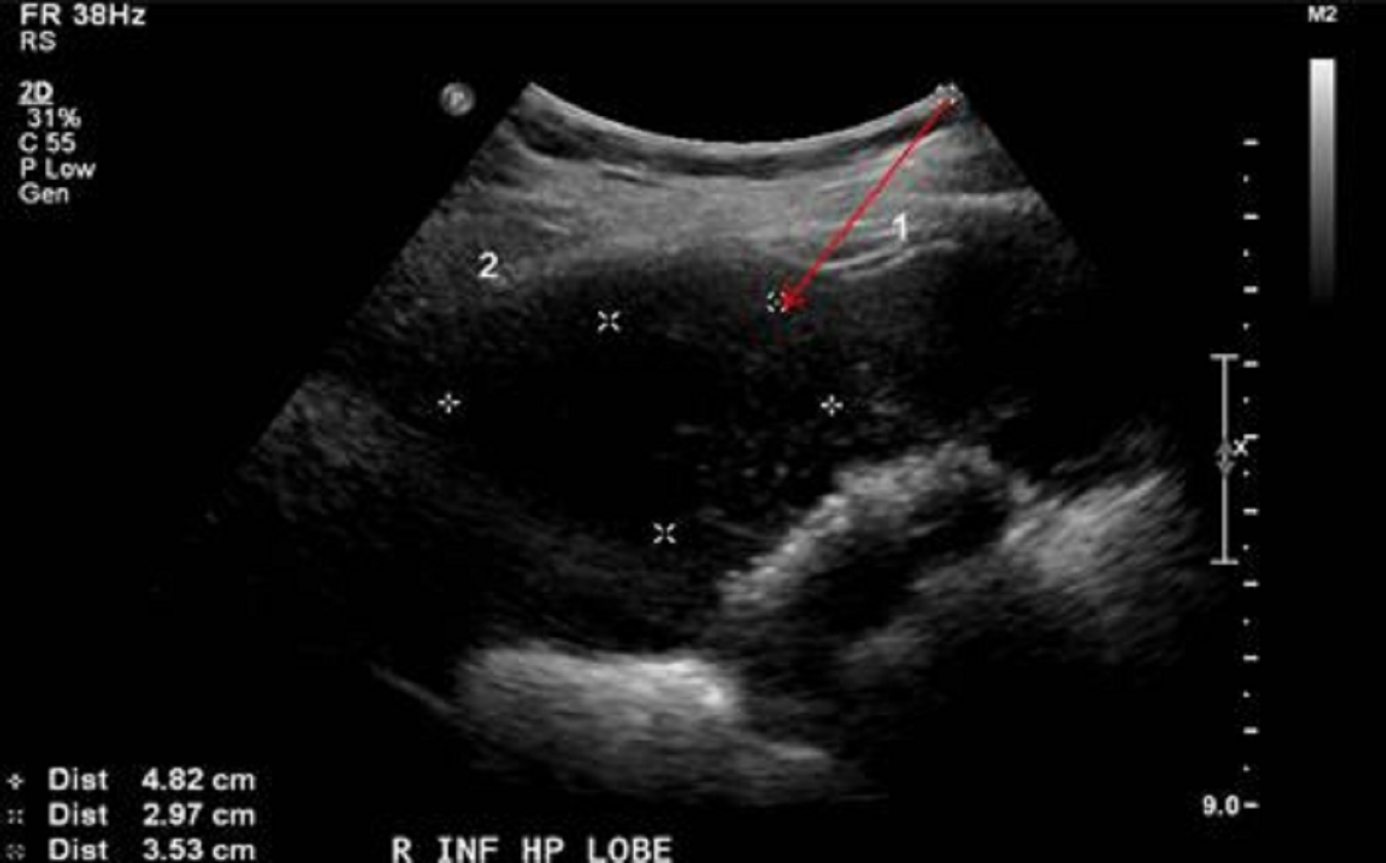
Preparatory imaging of ultrasound guided biopsy in the companion case. The image demonstrates the target lesion and the planned route of the needle (red arrow). The biopsy results revealed a primary biliary cancer, most likely gallbladder carcinoma.

The most common ultrasound findings include calcified and echogenic mucosal masses, which can be associated with cholelithiasis or porcelain gallbladder [15]. High-risk features on ultrasonography also include solitary or displaced gallstone, intraluminal mass, gallbladder-replacing or invasive mass, and discontinuity of the mucosal echo [15]. Other findings that are suggestive of GBC include the loss of the interface between the gallbladder and liver or direct liver infiltration [15]. Moreover, ultrasound abnormalities are often more subtle in early stage disease, making detection more challenging [15]. If abnormalities or suspicious findings are detected on ultrasound, further evaluation with other non-invasive imaging is warranted. Consideration of GBC in the differential diagnosis may help to improve detection before surgery (Figures 5-7), potentially leading to the discovery of the disease in its early stages.

The most useful, non-invasive imaging studies for evaluating GBC preoperatively include CT, magnetic resonance imaging (MRI), and/or magnetic resonance cholangiopancreatography (MRCP) (Figures 5, 6). CT has been shown to be useful in defining the extent of GBC and in determining the resectability in advanced stages [16-17]. Additionally, CT has a low to moderate sensitivity for detecting gastrointestinal, omental, and abdominal wall involvement, but because of its high positive predictive value in detecting liver invasion, lymph node involvement, and/or distant metastases, it remains a useful imaging study in GBC preoperatively [16-17]. MRI and MRCP have also been shown to be useful in preoperative staging of GBC with a high sensitivity in identifying hepatic invasion and lymph node metastasis [18]. Biopsies of the detected gallbladder masses are often performed via ultrasound guidance (Figure *7*), but can also be performed under CT guidance, endoscopic ultrasound guidance, endoscopic retrograde cholangiopancreatography (ERCP), or by laparoscopy.

Regarding further procedures and tests for GBC, endoscopic ultrasound (EUS) is a minimally invasive procedure that has been shown to be more accurate than RUQ ultrasound at imaging the gallbladder. EUS is also helpful in the differential diagnosis of gallbladder polyps and excellent in staging tumor depth [19-20]. ERCP and PET are two less commonly used imaging modalities used for evaluating GBC preoperatively. Generally, PET is not widely used prior to surgical resection because of its low sensitivity for detecting extrahepatic metastases, especially in patients with peritoneal carcinomatosis [21]. Although PET/CT is not commonly used before surgical resection, several studies have shown a potential benefit in detecting distant metastasis [21-24]. One retrospective study demonstrated that in patients with potentially resectable tumors based on conventional imaging, PET imaging was able to detect occult metastatic disease and ultimately changed the management in almost twenty-five percent of the patients [24].

## Conclusions

Our case illustrates the challenge of preoperative, early-stage GBC diagnosis. Despite multiple imaging studies, GBC was not diagnosed in the described patient until laparoscopy for acute cholecystitis. In the companion case, GBC was advanced at the time of imaging, which may have been due to patient denial of symptoms. Given that early detection is important in both decreasing the morbidity and mortality of GBC, with potential for surgical cure in cases limited to the gallbladder, patients with RUQ ultrasound findings such as gallstones or polyps should be offered surgical consultation. Further, consideration of GBC within the differential diagnosis by primary care clinicians, radiologists, and surgeons may serve to maximize discovery before the time of surgery.

"The views expressed in this case report are those of the authors and do not reflect the official policy of the Department of Army/Navy/Air Force, Department of Defense, or U.S. Government."
